# Characterization of the depolymerizing activity of commercial lipases and detection of lipase-like activities in animal organ extracts using poly(3-hydroxybutyrate-*co*-4-hydroxybutyrate) thin film

**DOI:** 10.1186/s13568-016-0230-z

**Published:** 2016-10-12

**Authors:** Pei-Shze Mok, Diana Hooi-Ean Ch’ng, Soo-Peng Ong, Keiji Numata, Kumar Sudesh

**Affiliations:** 1 School of Biological Sciences, Universiti Sains Malaysia, 11800 USM, Penang Malaysia; 2 Enzyme Research Team, RIKEN Center for Sustainable Resource Science, 2-1 Hirosawa, Wako, Saitama 351-0198 Japan

**Keywords:** P(3HB-*co*-4HB), Lipase, Lipase depolymerizing activity, Animal organ extract

## Abstract

Poly(3-hydroxybutyrate-*co*-4-hydroxybutyrate) [P(3HB-*co*-4HB)] is one of the polyhydroxyalkanoate (PHA) copolymers which can be degraded by lipases. In this study, the depolymerizing activity of different known commercial lipases was investigated via microassay using P(3HB-*co*-92 mol % 4HB) thin film as substrate. Non-enzymatic hydrolysis occurred under conditions in which buffers with pH 12 and 13 were added or temperature of 50 °C and above. Different concentrations of metal ions or detergents alone did not cause the film hydrolysis. The depolymerizing activity of lipases on P(3HB-*co*-4HB) was optimum in the pH range of 6–8 and at temperatures between 30 and 50 °C. Addition of metal ions and detergents in different concentrations was also shown to cause variable effects on the depolymerizing activity of commercial lipases. Pancreatic extracts from both mouse and chicken showed similar depolymerizing activity as the commercial lipases on the P(3HB-*co*-4HB) film. The presence of lipolytic enzymes in the organ extracts was confirmed with another lipase activity assay, *p*-nitrophenyl laurate assay. For the first time this has produced a direct evidence for the involvement of lipase-like enzymes from animal in the degradation of this PHA. Lipase is most likely the enzyme from pancreas that was involved in the degradation.

**Electronic supplementary material:**

The online version of this article (doi:10.1186/s13568-016-0230-z) contains supplementary material, which is available to authorized users.

## Introduction

Polyhydroxyalkanoate (PHA) is microbial polyester synthesized by various types of bacteria such as *Cupriavidus necator*, *Alcaligenes* spp., *Pseudomonas* spp., *Bacillus* spp., *Aeromonas hydrophila* and *Burkholderia sacchari* (Verlinden et al. 2007). Many studies are ongoing for the development of PHAs to replace some conventional plastics. Besides the potential use of PHAs as ecofriendly plastic materials, other types of niche applications for PHAs have also been proposed. For example, Sudesh et al. have proposed the use of PHA as cosmetic facial oil blotting film (Sudesh et al. 2007). Besides, PHA can also be used as a probe for the detection of microbial activities since it can be degraded by microbes via enzymatic reaction (Sudesh 2010). PHAs containing 4-hydroxybutyrate (4HB), 3-hydroxypropionate (3HP) or 5-hydroxyvalerate (5HV) share a unique property, whereby they are hydrolysable by the enzyme lipase (Mukai et al. 1993; Chuah et al. 2013). PHAs containing (4HB) monomer such as poly(4-hydroxybutyrate) [P(4HB)] and poly(3-hydroxybutyrate-*co*-4-hydroxybutyrate) [P(3HB-*co*-4HB)] are especially attractive (Martin and Williams 2003). They are widely known as bioabsorbable materials which have potential application in medical field as scaffold and suture. The physical and thermal properties have been studied as described in Additional file 1: Table S1 (Saito et al. 1996). Recently, this copolymer has also been used as substrate for the qualitative and quantitative analysis of lipase depolymerizing activity (Ch’ng and Sudesh 2013).

Lipases are known as glycerol ester hydrolases which act on the carboxyl ester bonds in triacylglycerols such as oils and fat to produce glycerol and free fatty acids (Li and Zhang 2005). They play a vital role in industrial field such as manufacturing of food and flavor, detergents, fine chemical, baking, leather and paper as well as bioremediation (Jaeger and Eggert 2002; Hasan et al. 2006). Lipases can be obtained from microorganisms, plants and animals (Jaeger et al. 1999). The hydrolytic activity of lipases is commonly measured by methods such as titrimetry and colorimetry (Stoytcheva et al. 2012). Besides all those methods, lipase depolymerizing activity can be readily analyzed as changes in the opacity of the P(3HB-*co*-4HB) thin film when lipase is in contact with the film. Changes on the transparent film of P(3HB-*co*-4HB) by lipases can be seen as opaque hydrolysis spots with naked eyes. The opacity can be further measured and correlated to concentration of the lipase (Ch’ng and Sudesh 2013). The image of the film with these hydrolysis spots is scanned using optical scanner in black background. The densities of hydrolysis spots are then quantified using a web-downloaded freeware (ImageJ) (Abràmoff et al. 2004). This measurement method is applied according to the method of measuring intensity of bands in gel electrophoresis (Gassmann et al. 2009). The relative densities are then calculated to reduce the background noise. This assay works based on the principle that lipase acts on the amorphous part of the P(3HB-*co*-4HB) film, spreading to the remaining crystalline part. Erosion on the P(3HB-*co*-4HB) film surface causes its opacity (Kumagai and Kanesawa 1992; Sudesh and Abe 2000). The thin film which is initially transparent becomes opaque due to the hydrolysis of amorphous regions leaving the crystalline regions which reflects light (Additional file 1: Figure S1).

In this study, P(3HB-*co*-4HB) thin film was characterized for microassay of commercial lipases and lipase-like activity in animal organ extracts. Only droplet of lipase solution was required for the depolymerizing activity on the film. By incubating the lipase droplet on the film, the depolymerizing activity can be readily analyzed as changes in the opacity of the thin film from transparent to opaque. We have further determined the factors that affect the lipase depolymerizing activity on the film. Factors such as pH, temperature and addition of metal ions and detergent on lipase depolymerizing activity were investigated. In addition, this method was used to detect the presence of lipase-like depolymerizing activity in animal organ extracts. It is demonstrated for the first time that there are enzymes that can degrade P(3HB-*co*-4HB) in animal organ extracts and these enzymes are most probably lipases.

## Materials and methods

### Source of commercial lipases

Commercial lipases of bacterial, fungal and animal sources were purchased from Sigma-Aldrich. They are lipases from *Candida antarctica*, *Candida rugosa*, *Mucor javanicus*, porcine pancreas, *Pseudomonas cepacia*, *Pseudomonas fluorescens*, *Rhizopus arrhizus*, *Rhizopus niveus* and *Rhizopus oryzae*. The sources of lipases and each concentration used in this study have been tabulated (Additional file 1: Table S2). Phosphate buffered saline (PBS), pH 7.4 was used to dissolve the lyophilized lipases.

### Biosynthesis, extraction and purification of P(3HB-*co*-4HB)

P(3HB-*co*-4HB) was biosynthesized by *Delftia acidovorans* JCM 10181 via two-stage cultivation as previously described (Lee et al. 2004). The bacterial cell was first cultivated in 2× nutrient broth (NB) medium at pH 7.0 supplemented by 1 % w/v of glucose for 24 h. The cell was then harvested and transferred aseptically into nitrogen-free mineral medium (NM) at pH 7.0. NM consisted of 0.37 g/L of K_2_HPO_4_, 0.58 g/L of KH_2_PO_4_, 0.1 mM of MgSO_4_·7H_2_O and 0.1 mL/L trace elements (TE) solution. TE solution consisted of 2.78 g/L of FeSO_4_·7H_2_O, 1.98 g/L of MnCl_2_·4H_2_O, 2.81 g/L of CoSO_4_·7H_2_O, 1.67 g/L of CaCl_2_·2H_2_O, 0.17 g/L of CuCl_2_·2H_2_O and 0.29 g/L of ZnSO_4_·7H_2_O in 0.1 M of HCl. In order to produce copolymer consisting 4HB, 1 % v/v of 1,4-butanediol was added as sole carbon source. The cell was harvested after 48 h and subjected to lyophilisation using freeze-drier (Labconco FreeZone). Gas chromatography (GC) analysis was carried out to determine PHA content and the monomer compositions (Braunegg et al. 1978). The cell was then stirred in chloroform with concentration of 1 g/150 mL to release P(3HB-*co*-4HB) from the cell. The cell debris was filtered using Whatman No. 1 filter paper and the polymer in chloroform was concentrated using Eyela rotary evaporator. P(3HB-*co*-4HB) was further precipitated using methanol by drop-wise method.

### Preparation of P(3HB-*co*-4HB) film

P(3HB-*co*-4HB) thin film was casted as previously described (Ch’ng and Sudesh 2013). A clean Petri dish (7 cm in diameter) was placed on a flat surface. P(3HB-*co*-4HB) was dissolved in chloroform (1 mg/mL) and poured into the Petri dish. The dish was covered with aluminum foil with 9 equal distance holes. The chloroform was allowed to evaporate for 1–2 days to form homogeneous and smooth surface of film.

### Qualitative and quantitative analysis of hydrolysis spot densities on P(3HB-*co*-4HB) film by lipases

Preparation of lipase samples as well as qualitative and quantitative analysis of hydrolysis spot densities were carried out as previously described (Ch’ng and Sudesh 2013). Different types of lipases were weighed and dissolved in PBS (pH 7.4) respectively. Triplicates of 10 µL lipase solution were dropped on P(3HB-*co*-4HB) film. Buffer without addition of lipase was used as negative control. The film with lipase droplets was incubated at 37 °C for 30 min. After that, the droplets were washed away by gently rinsing with distilled water and the film was allowed to dry. The image of the film was then scanned using optical scanner with a black background. The brightness and contrast of image was adjusted and the opaque spot formed on the P(3HB-*co*-4HB) film was cropped out, aligned and further analyzed using ImageJ, a freeware downloaded from internet. The color of the picture was inverted by changing the spot to black color with white background. The density of spot was measured and the average density was further calculated as relative density by dividing the density of sample by that of negative control.

### Observation of P(3HB-*co*-4HB) film surface changes after depolymerization by lipases via scanning electron microscope (SEM)

A cover slip (22 mm × 22 mm) was placed on a 7 cm glass Petri dish balanced on a flat surface. P(3HB-*co*-4HB) was dissolved in chloroform (1 mg/mL) and poured into the Petri dish. The Petri dish was then covered with aluminum foil and the solvent was evaporated to form a thin film. After that, the cover slips were lifted up with care without touching the surface of the film. Three different concentrations (0.5, 1.0 and 2.5 mg/mL) of lipase from *P*. *fluorescens* in PBS pH 7.4 were prepared. Further step was carried out as mentioned in qualitative and quantitative analysis. The film was then coated with gold for SEM observation. The surface morphologies of the P(3HB-*co*-4HB) film before and after incubation with lipase were observed using SEM (Leo Supra 50 VP Field Emission, Germany).

### Weight loss of P(3HB-*co*-4HB) via enzymatic hydrolysis

In order to measure the weight loss of P(3HB-*co*-4HB) caused by lipase hydrolysis, quartz crystal microbalance, QCM (AFFINIX Q4, Initiam, ULVAC, Kanagawa, Japan) was used according to a previous study (Numata et al. 2007). The oscillator which was 27 MHz AT-cut quartz crystal with deposition of Au electrodes on both sides was washed using 1:3 % v/v of hydrogen peroxide and sulphuric acid and followed by rinsing using Milli-Q water. P(3HB-*co*-4HB) was first dissolved in chloroform and casted by dropping 2 µL of 10 mg/mL of P(3HB-*co*-4HB) onto oscillator. The polymer was then air-dried for 1 day and fixed into QCM. The temperature of QCM was set as 37 °C. The oscillator with PHA film was flooded with 500 µL of PBS, pH 7.4 and stirred for approximately 30 min to stabilize the frequency. Lipase solution with specific concentration was added into the stirred buffer and the frequency change by time was recorded. The weight loss of polymer was then calculated based on change of frequency recorded by microcomputer system.

### Depolymerizing activity analysis of commercial lipases under different physical and chemical conditions

#### Effect of pH

Buffers at pH 1–13 were prepared according to Additional file 1: Table S3 and 10 µL of each buffer was dropped on the surface of P(3HB-*co*-4HB) film for further analysis as mentioned in qualitative and quantitative analysis. All types of lipases were weighed and dissolved in buffers with pH ranging from 1 to 11 (Lu et al. 2013). Qualitative and quantitative analysis were carried out.

#### Effect of temperature

PBS at pH 7.4 (10 µL) was dropped on the P(3HB-*co*-4HB) film and incubated for 30 min under different temperatures ranging from 15 to 60 °C using incubator shaker (IKA KS 4000 ic control) and the film was subjected to qualitative and quantitative analysis. For lipase depolymerizing activity assay, similar method was applied, except with the substitution of PBS by lipase solution. The polymer film was incubated for 30 min under different temperatures ranging from 15 to 45 °C.

#### Effect of metal ions

Different concentrations (1, 10 and 100 mM) of calcium chloride, copper (II) chloride, magnesium chloride, manganese (II) chloride and nickel (II) chloride were prepared respectively in PBS, pH 7.4 (Lu et al. 2013). They were first subjected to qualitative and quantitative analysis to screen for false positive. Then, final concentrations of 0.1, 1 and 10 mM of metal ions in lipase solutions were also subjected to qualitative and quantitative analysis. Lipase solution without addition of metal ions was used as positive control while buffer without any lipase and metal ions was used as negative control. The relative densities of hydrolysis spots were calculated by dividing the densities of hydrolysis spots formed by solutions with lipase and metal ion with those that only contain lipase.

#### Effect of detergents

Sodium dodecyl sulfate (SDS), Tween 20, Tween 80 and Triton X-100 with concentrations of 0.1, 1 and 10 % were prepared respectively (Lu et al. 2013). They were first subjected to qualitative and quantitative analysis to screen for false positive. Final concentrations of 0.01, 0.1 and 1 % of detergents in lipase solutions were subjected to qualitative and quantitative analysis. Lipase solution without addition of detergent solution was used as positive control while buffer without any lipase and detergent solution was used as negative control. The relative densities of hydrolysis spots were calculated by dividing the densities of hydrolysis spots formed by solutions with lipase and detergent with those that only contain lipase.

### Preparation of animal organ crude extracts for lipase-like depolymerizing activity assays

Mice (*Mus musculus*) used in this study were anesthetized by exposure to diethyl ether followed by cervical dislocation. They were then dissected using dissection equipments which were sterilized with ethanol. Duodenum, heart, kidney, liver, lung, pancreas, spleen and stomach of the mice were collected and washed with cold saline water containing 0.9 % sodium chloride. Wet weight of the organs was obtained and the organs were ground using mortar and pestle under cold condition. 10 mL of PBS (pH 7.4) was added to every 1 g of respective organ. The crude organ solution was centrifuged at 12000 rpm for 2 min and the supernatant was collected as crude extract for further analysis.

Chicken *(Gallus gallus domesticus*) organs such as duodenum, gizzard, liver, large intestine and small intestine were purchased freshly from the local market and stored in an ice box. Fat attached to the organs was also extracted for this analysis. The chicken organs were then processed in a similar manner as that of the mouse organs described above.

### Screening of depolymerizing activity of animal organ crude extracts using P(3HB-*co*-4HB) as substrate

Animal organ was heated at 95 °C for 30 min in order to denature the protein. Crude extract (20 µL) with and without heating were dropped on the surface of P(3HB-*co*-4HB) film respectively. The film was incubated at 37 °C for 60 min and any changes observed were recorded. The film was then washed and dried to remove the residual crude extract. After that, the film was subjected to qualitative and quantitative analysis.

### Screening of lipase-like hydrolytic activity of animal organ crude extracts using *p*NPL as substrate

Standard curve was first constructed using *p*-nitrophenol (Kilcawley et al. 2002). Substrate emulsion was prepared by mixing 90 mL of 0.1 M phosphate buffer at pH 7.4 with 0.1 % w/v polyvinyl alcohol (PVA), 0.9 % w/v Triton-X, and 5 mM *p*NPL that were dissolved in 10 mL of dimethyl sulfoxide (DMSO). The crude extract from each animal organ was centrifuged at 12,000 rpm for 2 min and the supernatant (0.1 mL) was added into 1.9 mL of emulsion and subsequently incubated for 10 min at 37 °C. The absorbance of the mixture was recorded using spectrophotometer at a wavelength of 410 nm. Each assay was conducted in triplicate. The amount of *p*-nitrophenol released was calculated by using the standard curve of *p*-nitrophenol concentration versus absorbance. The lipase activity of the sample solution from crude extract using *p*NPL as substrate was obtained using the equation below.

$${\text{Lipase}}\; {\text{activity}} \;(\upmu {\text{mol}} /{\text{mL }}\;\text{min} ) = \frac{{{\text{Amount of}} \,p{-}{\text{nitrophenol released}} \,(\upmu {\text{mol}})}}{{{\text{volume of enzyme in assay mixture}} \,({\text{mL}}) \times {\text{Incubation time}} \,\left( {\text{min} } \right)}}$$

### Statistical analysis

The densities of hydrolysis spots produced on P(3HB-*co*-4HB) film under different conditions were analyzed by analysis of variance using general linear model (GLM) procedures. Means were compared using Tukey’s HSD at p < 0.05.

## Results

### Surface changes of P(3HB-*co*-4HB) film after depolymerization by lipases

**Fig. 1:**
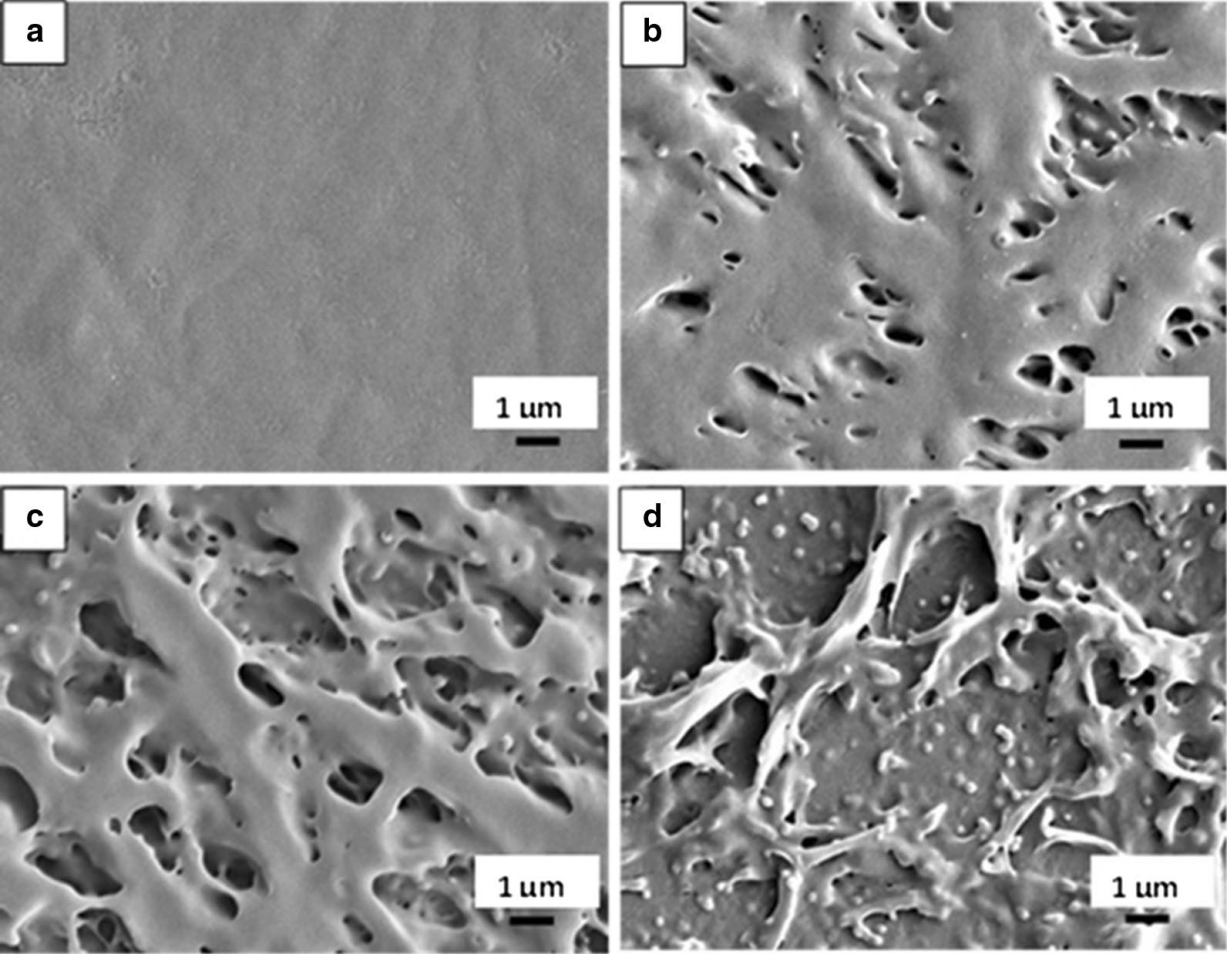
SEM micrographs of P(3HB-*co*-4HB) film after incubation with buffer and lipase solutions. SEM micrographs of P(3HB-*co*-4HB) film after incubation with 10 μL of **a** PBS pH 7.4, **b** lipase at 0.5 mg/mL from *P*. *fluorescens* in PBS pH 7.4, **c** lipase at 1.0 mg/mL from *P*. *fluorescens* in PBS pH 7.4 and **d** lipase at 2.5 mg/mL from *P*. *fluorescens* in PBS pH 7.4 for 30 min at 37 °C

Surface changes on P(3HB-*co*-4HB) film after being degraded by lipases was observed via SEM. PBS did not cause any changes to the film, so the film surface remained smooth (Fig. 1a). However, after incubation with lipases with concentrations of 0.5, 1.0 and 2.5 mg/mL, holes were produced on the film causing it to be rough. When the lipase concentration was increased, more holes were produced on the film. The film surface became rougher when higher concentration of lipase was used (Fig. 1b–d). There was also clear observation using naked eyes whereby opaque spot was only formed on the film when there was lipase activity. As the lipase activity increased, the opacity of the spots also increased.

### QCM measurement on weight loss of P(3HB-*co*-4HB) via enzymatic hydrolysis

The oscillator with P(3HB-*co*-4HB) film was stabilized for around 30 min. According to the results recorded by microcomputer system, the frequency decreased for few minutes due to increase of weight after addition of lipase solution. Then, frequency started to shift upward as the lipase solution started to hydrolyze P(3HB-*co*-4HB) and in turn caused weight loss (data not shown). Relationship between weight loss of P(3HB-*co*-4HB) and relative density of hydrolysis spots on P(3HB-*co*-4HB) was constructed based on Additional file 1: Figure S2a and b. As the weight loss of P(3HB-*co*-4HB) increased, the relative density of hydrolysis spot also increased. These results indicate that the relative density of hydrolysis spot was proportional towards the weight loss of P(3HB-*co*-4HB).

### Effect of pH on P(3HB-*co*-4HB) film and lipase depolymerizing activity

When buffers with pH 1–13 were dropped on the surface of P(3HB-*co*-4HB) film, droplets formed on the film were all spherical in shape prior to incubation except for buffer with pH 13. The droplets of buffer with pH 13 started to disperse as they were dropped on P(3HB-*co*-4HB) film. After incubation for 30 min, droplets of pH 12 buffer were also dispersed. Besides, faint hydrolysis spots were formed on the film. However, buffers with pH from 1 to 11 did not cause any changes to the film surface after incubation. Droplets of lipase solutions with pH ranging from 1 to 11 were then screened using P(3HB-*co*-4HB) film as substrate. Densities of hydrolysis spots produced on the film surface were calculated and the relative densities of hydrolysis spots are shown in Table 1. Most of the lipases produced hydrolysis spots with higher densities at pH 6–8. Some lipases were able to form hydrolysis spots in a wide pH range. For example, the pH range for lipase from *P*. *cepacia* for screening lipase depolymerizing activity using this polymer film was from pH 4–11. Hydrolysis spots with the highest relative densities were formed by this lipase at pH 11. However, the droplets formed were dispersed and the hydrolysis spots were not consistent in size. Thus, results were not recorded for the spot densities.

**Table 1:** Relative densities of hydrolysis spots produced by commercial lipases at pH 1–11 on P(3HB-*co*-4HB)

pH	Relative densities of hydrolysis spots produced by lipase from different sources								
	*C*. *antarctica*	*C*. *rugosa*	*M*. *javanicus*	Porcine pancreas	*P*. *cepacia*	*P*. *fluorescens*	*R*. *arrhizus*	*R*. *niveus*	*R*. *oryzae*
Buffer	1.00 ± 0.30^a^	1.00 ± 0.15^a^	1.00 ± 0.32^a^	1.00 ± 0.11^a^	1.00 ± 0.15^a^	1.00 ± 0.27^a^	1.00 ± 0.18^a^	1.00 ± 0.20^a^	1.00 ± 0.21^a^
1	0.70 ± 0.87^a^	0.82 ± 0.24^a^	1.05 ± 0.30^a^	0.84 ± 0.77^a^	0.72 ± 0.49^a^	1.26 ± 0.79^a^	5.77 ± 0.42^abc^	3.90 ± 0.21^ab^	1.19 ± 0.39^a^
2	1.35 ± 0.41^a^	0.89 ± 0.26^a^	1.03 ± 0.20^a^	0.55 ± 0.50^a^	0.88 ± 1.28^a^	1.09 ± 0.45^a^	5.53 ± 0.57^abc^	2.03 ± 0.32^a^	1.15 ± 0.14^a^
3	25.98 ± 0.30^d^	0.80 ± 0.11^a^	1.10 ± 0.55^a^	1.04 ± 0.47^a^	2.53 ± 0.15^a^	1.28 ± 0.91^a^	6.03 ± 0.53^abc^	6.41 ± 0.54^abc^	1.19 ± 0.29^a^
4	14.27 ± 0.20^bc^	4.24 ± 0.26^b^	0.83 ± 0.28^a^	0.91 ± 0.65^a^	15.51 ± 0.40^abcd^	2.29 ± 0.30^ab^	27.65 ± 0.49^bcd^	8.24 ± 0.23^bcd^	1.20 ± 0.20^a^
5	20.93 ± 0.25 ^cd^	6.11 ± 0.09^c^	3.56 ± 0.42^ab^	0.81 ± 0.53^a^	19.05 ± 0.10^bcd^	4.36 ± 0.34^ab^	26.77 ± 0.59^bcd^	11.51 ± 0.36^cde^	1.20 ± 0.16^a^
6	30.66 ± 0.30^d^	7.26 ± 0.05^c^	3.78 ± 0.39^ab^	5.30 ± 0.24^bc^	17.47 ± 0.25^abcd^	4.48 ± 0.28^ab^	29.37 ± 0.04 ^cd^	13.35 ± 0.13^de^	5.19 ± 0.60^b^
7	51.64 ± 0.01^e^	6.77 ± 0.09^c^	8.35 ± 0.41^b^	8.91 ± 0.36^c^	20.85 ± 0.20 ^cd^	7.45 ± 0.48^abc^	23.60 ± 0.77^abcd^	14.79 ± 0.26^e^	3.77 ± 0.40^ab^
8	6.40 ± 0.39^ab^	0.83 ± 0.33^a^	6.53 ± 0.69^b^	8.58 ± 0.39^c^	11.45 ± 0.31^abc^	17.59 ± 0.30^c^	35.18 ± 0.22^d^	17.34 ± 0.03^e^	5.20 ± 0.15^b^
9	1.20 ± 0.23^a^	0.71 ± 0.22^a^	0.91 ± 0.29^a^	3.86 ± 0.22^ab^	10.15 ± 0.26^abc^	17.26 ± 0.25^c^	24.28 ± 0.20^abcd^	1.38 ± 0.42^a^	6.69 ± 0.08^b^
10	0.99 ± 0.27^a^	0.80 ± 0.13^a^	0.88 ± 0.55^a^	1.32 ± 0.41^a^	8.27 ± 0.02^abc^	18.26 ± 0.41^c^	3.15 ± 0.31^ab^	1.27 ± 0.25^a^	1.17 ± 0.35^a^
11	0.86 ± 0.38^a^	0.66 ± 0.30^a^	1.10 ± 0.62^a^	0.49 ± 0.53^a^	30.88 ± 0.57^d^	13.10 ± 0.57^bc^	3.00 ± 0.11^ab^	1.56 ± 0.47^a^	1.08 ± 0.24^a^

Buffer indicates PBS pH 7.4 without addition of lipase. The data are mean of triplicate. Mean data accompanied by different superscript alphabets are significantly different (Tukey’s HSD test, p < 0.05). The assay was conducted at 37 °C for 30 min

### Effect of temperature on P(3HB-*co*-4HB) film and lipase depolymerizing activity

The depolymerizing activities of lipases under different temperatures were also screened using P(3HB-*co*-4HB) film as substrate. Prior to the analysis with enzymes, PBS at pH 7.4 which acted as negative control was screened for its effect on film surface under different temperatures (Additional file 1: Figure S3). The buffer did not cause any changes to the film surface from 15 to 45 °C but hydrolysis spots were formed from 50 to 60 °C. Therefore, temperatures of 50 °C and above were not used in the following studies with lipases. Based on Table 2, most of the lipases tested formed opaque spots with higher densities from 30 to 45 °C while lower densities were detected for temperatures from 15 to 25 °C. Although some of the lipases showed increasing trend in hydrolysis spots densities as the temperature increased, further tests were not conducted for temperature above 50 °C due to the temperature limit of the film.

**Table 2:** Relative densities of hydrolysis spots produced by commercial lipases at different temperatures on P(3HB-*co*-4HB)

Temp. (°C)	Relative densities of hydrolysis spots produced by lipase from different sources								
	*C*. *antarctica*	*C*. *rugosa*	*M*. *javanicus*	Porcine pancreas	*P*. *cepacia*	*P*. *fluorescens*	*R*. *arrhizus*	*R*. *niveus*	*R*. *oryzae*
Buffer	1.00 ± 0.24^a^	1.00 ± 0.08^a^	1.00 ± 0.22^a^	1.00 ± 0.29^a^	1.00 ± 0.17^a^	1.00 ± 0.18^a^	1.00 ± 0.02^a^	1.00 ± 0.14^a^	1.00 ± 0.17^a^
15	10.04 ± 0.33^ab^	2.42 ± 0.58^ab^	1.07 ± 0.21^a^	1.25 ± 0.18^a^	2.15 ± 0.17^abc^	4.62 ± 0.10^bc^	4.69 ± 0.28^ab^	3.80 ± 0.23^b^	1.92 ± 0.32^a^
20	11.73 ± 0.28^bc^	2.73 ± 0.33^abc^	1.13 ± 0.50^a^	1.37 ± 0.41^a^	2.02 ± 0.14^ab^	3.07 ± 0.31^b^	11.10 ± 0.30^bc^	3.24 ± 0.08^b^	2.07 ± 0.26^a^
25	16.18 ± 0.36^bc^	3.93 ± 0.56^abcd^	4.41 ± 0.34^ab^	1.30 ± 0.38^a^	2.33 ± 0.34^abc^	3.72 ± 0.21^b^	8.99 ± 0.18^b^	4.60 ± 0.23^b^	3.24 ± 0.48^b^
30	17.94 ± 0.11^bc^	3.36 ± 0.33^abc^	4.52 ± 0.07^ab^	3.17 ± 0.30^ab^	5.28 ± 0.25 ^cd^	3.60 ± 0.33^b^	19.79 ± 0.23^d^	6.95 ± 0.10^c^	2.60 ± 0.07^b^
35	20.66 ± 0.04^c^	5.32 ± 0.15^bcd^	10.29 ± 0.52^bc^	4.16 ± 0.29^b^	7.72 ± 0.30^d^	6.60 ± 0.14^c^	24.54 ± 0.02^d^	7.20 ± 0.11^c^	7.25 ± 0.11^b^
40	17.58 ± 0.18^bc^	5.61 ± 0.11 ^cd^	8.34 ± 0.69^abc^	7.89 ± 0.18^c^	8.14 ± 0.14^d^	10.62 ± 0.03^d^	18.26 ± 0.27 ^cd^	12.66 ± 0.04^d^	8.30 ± 0.14^b^
45	14.63 ± 0.29^bc^	6.66 ± 0.08^d^	15.12 ± 0.06^c^	8.47 ± 0.24^c^	5.01 ± 0.17^bcd^	8.78 ± 0.08^d^	23.09 ± 0.04^d^	11.70 ± 0.10^d^	6.63 ± 0.22^b^

Buffer indicates PBS pH 7.4 without addition of lipase. The data are mean of triplicate. Mean data accompanied by different superscript alphabets are significantly different (Tukey’s HSD test, p < 0.05). The assay was conducted for 30 min

### Effect of metal ions on P(3HB-*co*-4HB) film and lipase depolymerizing activity

**Fig. 2:**
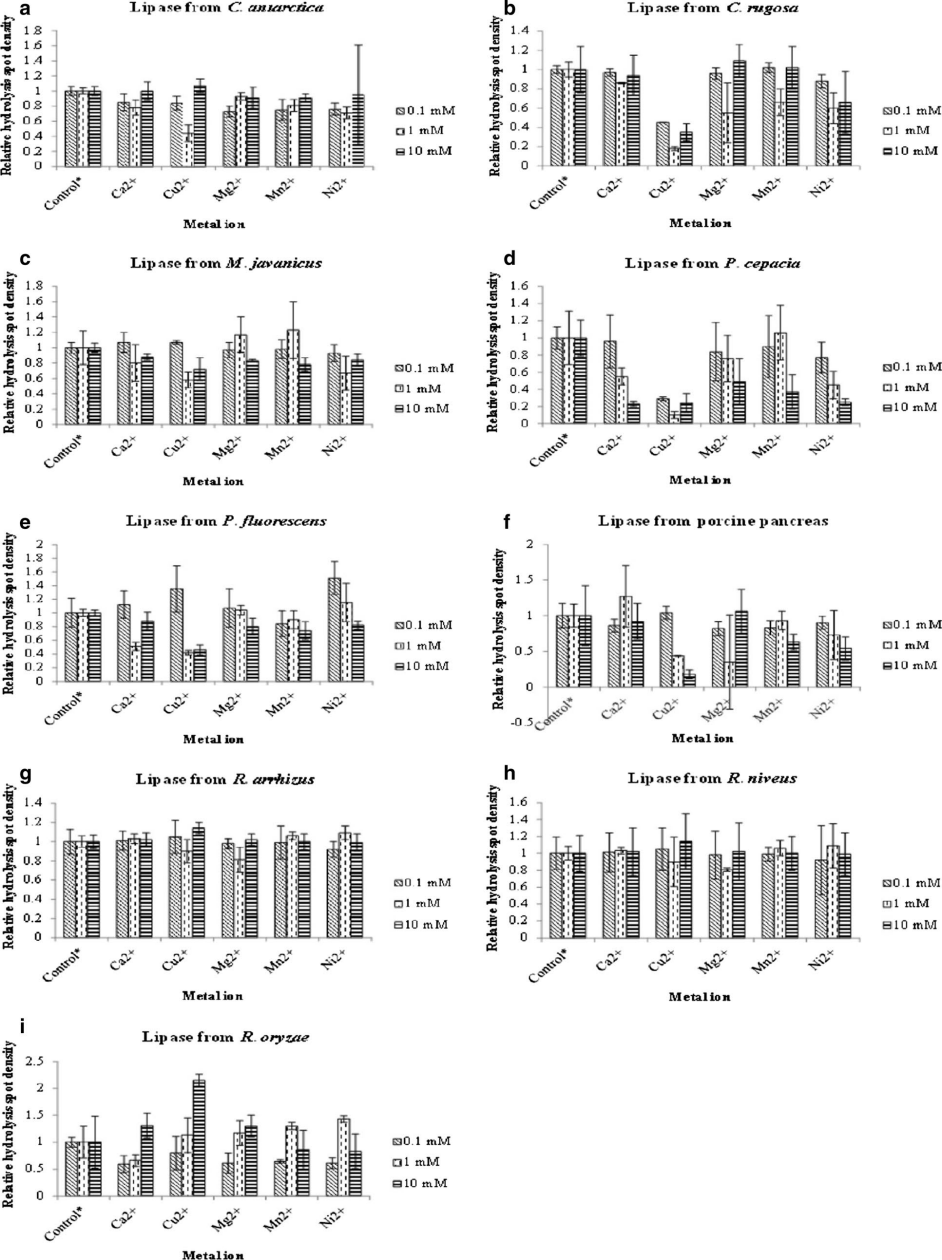
Hydrolysis spot densities of lipases after addition of metal ions with different concentration. Hydrolysis spot densities of lipase from **a** *C*. *antarctica*; **b** *C*. *rugosa*; **c** *M*. *javanicus;* **d** *P*. *cepacia;* **e** *P*. *fluorescens;* **f** Porcine pancreas; **g** *R*. *arrhizus;* **h** *R*. *niveus;* **i** *R*. *oryzae* after addition of metal ions with different concentration. Control *asterisk* represents lipase solution without addition of metal ions. The assay was conducted at 37 °C for 30 min. The data are mean of triplicate

The effect of metal ions on lipase depolymerizing activity using P(3HB-*co*-4HB) film as substrate was studied. Prior to the study, the metal ions were first screened for their effect on P(3HB-*co*-4HB) film. Different metal ions (Ca^2+^, Cu^2+^, Mg^2+^, Mn^2+^ and Ni^2+^) with concentrations of 0.1, 1 and 10 mM respectively were dropped on the film surface. After incubation for 30 min at 37 °C, no hydrolysis spot was formed on the film (figure not shown). Therefore, it can be hypothesized that these metal ions did not cause physical change or false positive result on the film. Next, the depolymerizing activities of lipase solution with and without the addition of metal ions were compared. Results have shown that 1 mM of metal ions either promoted or inhibited lipase depolymerizing activities while 0.1 mM and 10 mM did not show any effect on the lipase depolymerizing activity (Fig. 2). Interestingly, only lipase activity from *P*. *cepacia* was affected by 0.1 mM of Ca^2+^. Activity of this lipase was also reduced by 0.1 mM of Mg^2+^, Mn^2+^ and Ni^2+^. On the other hand, 1 mM of Mg^2+^ and Mn^2+^ increased the lipase activity of *M*. *javanicus* whereas Ca^2+^ with the same concentration increased the densities of hydrolysis spots produced by lipase from porcine pancreas. For lipase from *R*. *niveus*, 1 mM of Cu^2+^, Mg^2+^, Mn^2+^ and Ni^2+^ increased its lipase activity on the film. When the concentration of metal ions was increased to 10 mM, Cu^2+^ completely inhibited the lipase activity of *C*. *rugosa*, porcine pancreas and *P*. *fluorescens* but stimulated the lipase activity of *R*. *niveus* and *R*. *oryzae*. For lipase from *M*. *javanicus*, 10 mM of metal ions reduced the lipase activity on the film. Metal ions at this concentration also inhibited the activity of lipase from *P*. *cepacia*.

### Effect of detergents on P(3HB-*co*-4HB) film and lipase depolymerizing activity

**Fig. 3:**
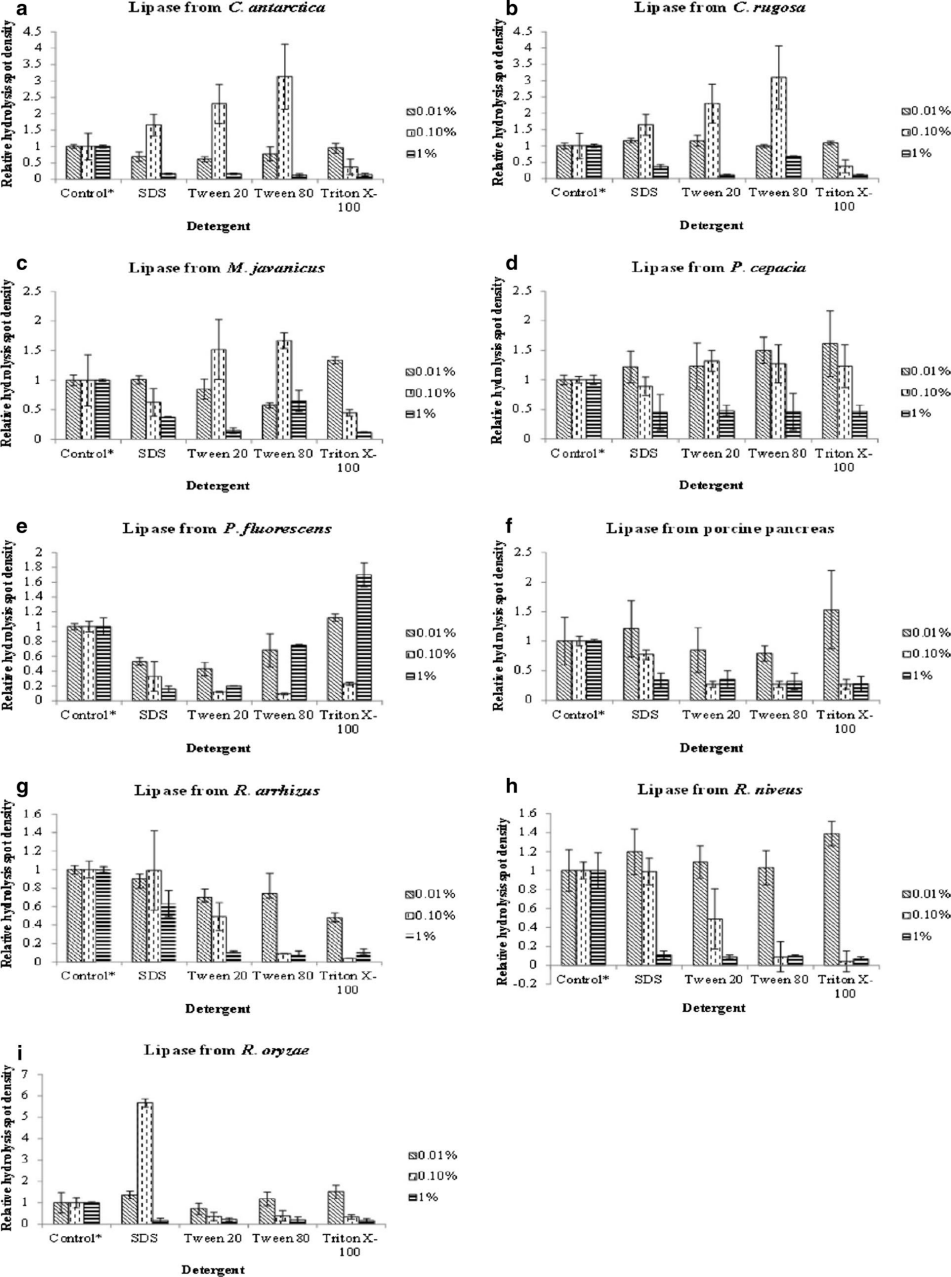
Hydrolysis spot densities of lipases after addition of detergents with different concentration. Hydrolysis spot densities of lipase from **a** *C*. *antarctica*; **b** *C*. *rugosa*; **c** *M*. *javanicus;* **d** *P*. *cepacia;* **e** *P*. *fluorescens;* **f** Porcine pancreas; **g** *R*. *arrhizus;* **h** *R*. *niveus;* **i** *R*. *oryzae* after addition of detergents with different concentration. Control *asterisk* represents lipase solution without addition of detergent. The assay was conducted at 37 °C for 30 min. The data are mean of triplicate

When detergents (SDS, Tween 20, Tween 80 and Triton X-100) at different concentrations were dropped on a polymer film for incubation, no hydrolysis spot was formed on the film, which eliminates the possibility of false positive result using these detergents (figure not shown). 0.01 % of detergents did not produce much effect on most of the commercial lipases but 0.1 % of detergents either increased or inhibited most of the lipases (Fig. 3). Detergents with concentrations of 1 % inhibited most of the lipase depolymerizing activity by formation of no or faint hydrolysis spots on the film. For lipase from *C*. *rugosa*, depolymerization was not affected by 0.01 % of detergents whereas depolymerization was completely inhibited by 0.1 % of Triton X-100. Interestingly, 0.1 % of SDS, Tween 20 and Tween 80 had increased the lipase depolymerizing activity. No hydrolysis spot was formed on the film after 1 % of Tween 20 and Triton X-100 were added into the lipase respectively while 1 % of SDS and Tween 80 had decreased the lipase depolymerizing activity on the film. Besides that, 0.01 % of Triton X-100 and 0.1 % of Tween 20 and Tween 80 increased lipase depolymerizing activity of lipase from *M*. *javanicus* while others inhibited its lipase activity. Both 0.01 and 0.1 % of detergents did not affect the lipase depolymerizing activity of lipases from porcine pancreas and *P*. *capacia* while 1 % of detergents inhibited its activity. Detergents at different concentrations completely inhibited lipase depolymerizing activity of lipase from *P*. *fluorescens* except that 1 % of Triton X-100 increased its lipase depolymerizing activity. For lipases from *Rhizopus* species, 0.1 and 1 % of detergents inhibited the lipase depolymerizing activity except that 0.1 % of SDS increased the activity of lipase from *R*. *oryzae*.

### Screening of depolymerizing activity in animal organ crude extracts using P(3HB-*co*-4HB) as substrate

Lipase from *P*. *cepacia* was used as positive control to compare the depolymerizing activity of the organ extracts from mouse and chicken. As can be seen from Additional file 1: Figures S4 and S5, duodenum and pancreatic extracts from both animals formed hydrolysis spots on P(3HB-*co*-4HB) film. Extracts of gizzard and fat from chicken produced faint hydrolysis spots on the film. Extracts from other organs did not form hydrolysis spots on the film. The animal organ extracts were also heated at 95 °C and screened using polymer film as substrate. No hydrolysis spot was formed on the film by the heated crude extracts.

**Fig. 4:**
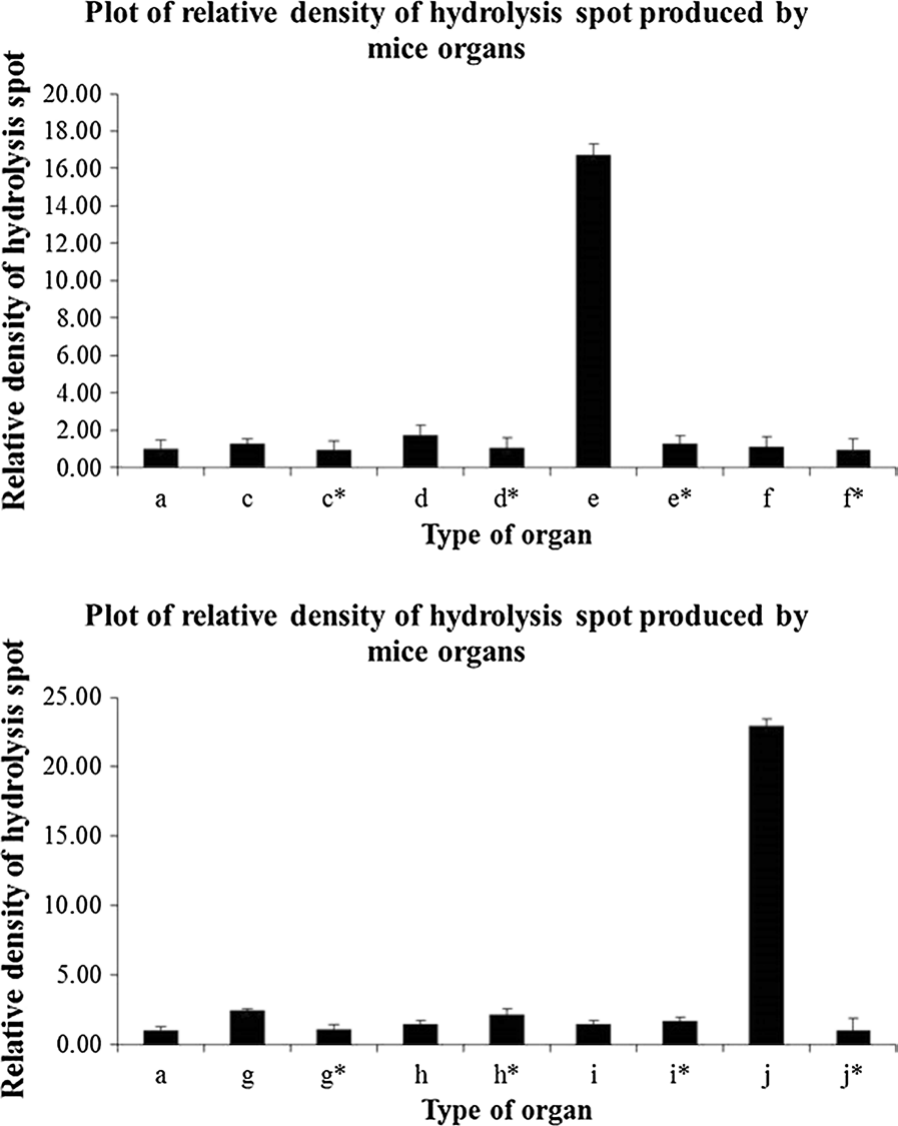
Hydrolysis spot densities produced by supernatant from mice organs. Hydrolysis spot densities produced by supernatant from mice organs which are *c* heart, c* heated heart, *d* stomach, d* heated stomach, *e* duodenum, e* heated duodenum, *f* kidney, f* heated kidney, *g* lung, g* heated lung, *h* liver, h* heated liver, *i* spleen, i* heated spleen, *j* pancreas, j* heated pancreas. *a* indicates PBS pH 7.4 which act as negative control. The assay was carried out at 37 °C for 1 h

**Fig. 5:**
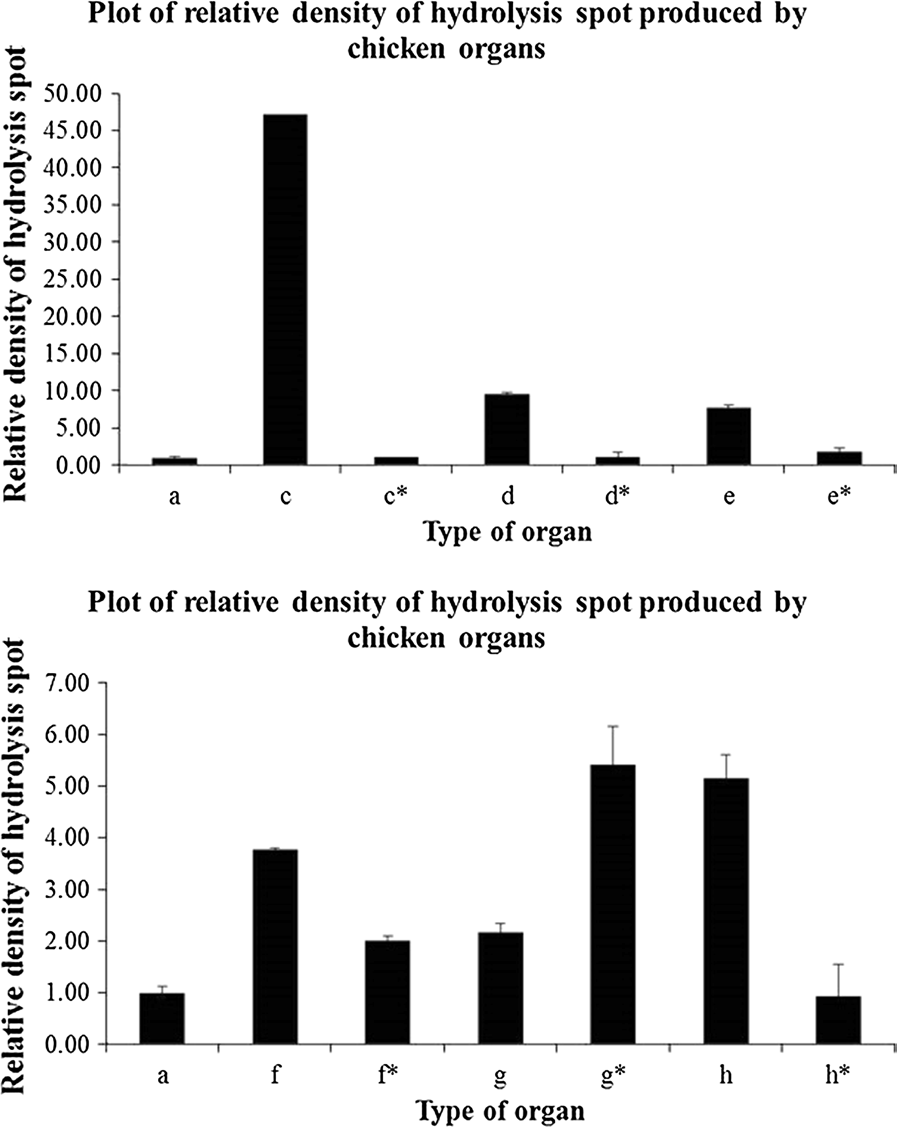
Hydrolysis spot densities produced by supernatant from chicken organs. Hydrolysis spot densities produced by supernatant from chicken organs which are *c* duodenum and pancreas, c* heated duodenum and pancreas, *d* fat, *d* heated fat, *e* gizzard, e* heated gizzard, *f* liver, f* heated liver, *g* large intestine, g* heated large intestine, *h* small intestine, h* heated small intestine. *a* indicates PBS pH 7.4 which act as negative control. The assay was carried out at 37 °C for 1 h

After screening the depolymerizing activity of organ crude extract, the density of hydrolysis spots was measured and its activity was calculated. Based on the results shown in Fig. 4, duodenum and pancreatic extracts from mice formed hydrolysis spots with higher densities compared to other mice organs that showed no significant difference with PBS pH 7.4 in their densities based on statistical analysis. From Fig. 5, duodenum and pancreatic extracts from chicken showed significant densities on the film. Gizzard and fat extracts also formed hydrolysis spots on the film but the densities of hydrolysis spots were lower compared to those of the hydrolysis spots produced by chicken’s duodenum and pancreatic extracts.

### Screening of lipase activity in animal organs crude extract using *p*NPL as substrate

**Fig. 6:**
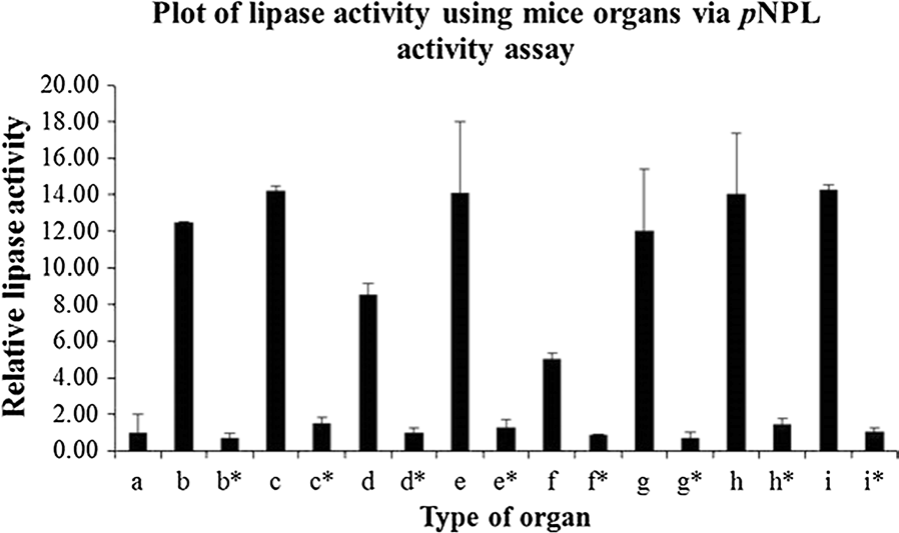
Lipase activities of mice organs using *p*NPL as substrate. The organs are *b* duodenum, b* heated duodenum, *c* liver, c* heated liver, *d* spleen, d* heated spleen, *e* heart, e* heated heart, *f* pancreas, f* heated pancreas, *g* stomach, g* heated stomach, *h* lungs, h* heated lungs, *i* kidney, i* heated kidney. *a* indicates PBS pH 7.4 which act as negative control. The assay was carried out at 37 °C for 10 min and the absorbance was taken at 410 nm. Data are mean of triplicate

**Fig. 7:**
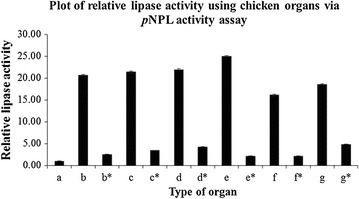
Lipase activities of chicken organs using *p*NPL as substrate. The organs are *b* duodenum and pancreas, b* heated duodenum and pancreas, *c* fat, c* heated fat, *d* gizzard, d* heated gizzard, *e* liver, e* heated liver, *f* large intestine, f* heated large intestine, *g* small intestine, g* heated small intestine. *a* indicates PBS pH 7.4 which act as negative control. The assay was carried out at 37 °C for 10 min and the absorbance was taken at 410 nm. Data are mean of triplicate

The crude extracts from animal organs were also screened for lipase activity using *p*NPL as substrate. Figures 6 and 7 show the lipase activities of supernatants from mouse and chicken organs using *p*NPL as substrate. Supernatants from all the mice organs showed significant lipase activity in this assay. In this case, duodenum showed lower lipase activity compared to other organs. The heated supernatants from mice organs did not show significant lipase activity using *p*NPL as substrate. All the extracts from chicken organs also showed significant lipase activity when assayed with *p*NPL. However, the heated crude extracts showed lower lipase activity compared to those without heating. Liver extract showed the highest lipase activity compared to lipase from *P. cepacia*.

## Discussion

Lipases are widely used in different applications such as detergents, cosmetics, food flavorings, diesels, papers and pulps. Thus, measurement of lipase activity is an important process for the quantification of lipases. Different from other reported lipase activity assays, P(3HB-*co*-4HB) is an eco-friendly option for lipase assay substrate as it is readily biodegradable. This copolymer was developed to screen for lipase activity by casting the polymer as a thin film. Lipase activity of commercial lipases had been screened and quantified using the thin polymer film as substrate (Ch’ng and Sudesh 2013). Further study on this polymer film is required to gain more insights of the factors which affect lipase depolymerizing activity on P(3HB-*co*-4HB) film. Herein, the depolymerizing activity of commercial lipases under different conditions was investigated.

Nine different commercial lipases were screened on P(3HB-*co*-4HB) film under different conditions and concentrations. Each lipase has its own optimum concentration where it is able to produce hydrolysis spots with the highest density on the film (Ch’ng and Sudesh 2013). The hydrolysis spots formed on the film were observed using SEM to verify the changes of film surface after incubation with lipase solutions. PBS that acted as negative control did not produce any change on the film surface as the film surface remained smooth. The film became rougher with increasing concentration of lipase and more holes were formed on the film due to higher rate of polymer degradation. The SEM analysis clearly revealed that the changes to the film surface were due to the enzyme but not the buffer.

The properties of lipase from various sources are unique and this can affect their hydrolytic activities. Although the production of lipase is dependent on the pH and temperature of growth for the host organism, lipase is active in wide range of pH and temperature (Gupta et al. 2004). Each lipase has its own optimum pH and temperature for the formation of hydrolysis spots on P(3HB-*co*-4HB) film. Before the effect of pH on depolymerizing activity was screened on P(3HB-*co*-4HB) film, buffers at different pH were screened to prevent formation of false positive results. The results showed that buffer at pH 13 dispersed after incubating the film while buffer at pH 12 formed faint hydrolysis spots on the film. These buffers at pH 12 and pH 13 were not suitable to be used for screening lipase depolymerizing activity. The dispersion of buffer at pH 13 showed that they did not form hydrophobic interaction with the P(3HB-*co*-4HB) film and thus, lipase activity could not be qualified and quantified accurately. According to Li et al. hydrophobic surface could be wetted after addition of acid or alkali by decreasing the water angle towards the hydrophobic surface (Li et al. 2006). For buffer at pH 12, non-enzymatic reaction was formed by this buffer on the film and thus lipase activity would be interfered by this result. Ginde and Gupta had reported that degradation of poly(glycolic acid) could be accelerated using alkaline buffer solution compared to neutral and slightly acidic solutions (Ginde and Gupta 1987). The depolymerizing activities of nine commercial lipases were then screened on P(3HB-*co*-4HB) film from pH 1–11. Each of the lipase was dissolved in buffer with different pH and they were found to work differently over a wide pH range to produce hydrolysis spots on the film. All the tested lipases were able to form hydrolysis spots at pH 6 and 7. At pH 8, all the tested lipases showed depolymerizing activity on the film except for the lipase from *C*. *rugosa*. Results from this study showed that pH 6–8 were the suitable pH range for the lipases to cleave ester bonds in P(3HB-*co*-4HB) polymer. This finding correlates well with reports of conventional lipase activity quantitative analysis by using substrates such as triacetin, triolein, tributyrin and olive oil with optimum pH of 7–8 (Gupta et al. 2003).

Besides pH, temperature is also an important factor that affects enzyme activity (Gupta et al. 2004). In addition, temperature also affects the state of a polymer, which in turn will cause non-enzymatic hydrolytic reactions. Previous report showed that higher temperature would decrease the molecular weight of PHA (Bonartsev et al. 2012). Therefore, the effect of temperature on polymer films and lipase depolymerizing activities was studied. It was found that when incubated at temperature of 50 °C and above, non-enzymatic hydrolytic degradation occurred on the P(3HB-*co*-4HB) film and formed opaque hydrolysis spots. According to the previous report, P(3HB-*co*-4HB) with more than 90 mol % of 4HB composition had melting temperature of about 50 °C (Saito et al. 1996). According to Doi and his colleagues, P(3HB-*co*-27 mol % 4HB) had higher molecular weight loss at 55 °C compared to P(3HB-*co*-17 mol % 4HB) due to random chain scission even though the weight of both copolymers was not reduced (Doi et al. 1990). It is believed that random chain scission occurred when P(3HB-*co*-4HB) was incubated with buffer droplets at temperatures above 50 °C and caused the formation of hydrolysis spots. Therefore, at this temperature, buffer is able to cause the change of physical appearance on the film. On the other hand, all the lipases were active over a wide range of temperatures and formed hydrolysis spots at above 30 °C. Lipases from *C*. *rugosa*, *M*. *javanicus*, porcine pancreas and *R*. *arrhizus* formed hydrolysis spots with the highest opacity at 45 °C. However, due to non-enzymatic degradation of the film at temperatures above 50 °C, lipase activity could not be tested at higher temperatures. It was found that assay of lipase activity on this film were most suitable at temperatures between 30 and 40 °C.

Metal ions are known to be able to stimulate or inhibit lipase activity without any cofactor (Gupta et al. 2004). According to the results of this study, most of the lipases tested were not influenced by 0.1 mM and 10 mM of metal ions. The concentration of metal ions at 0.1 mM might be too low to influence the lipase activity. Lu et al. had reported that metal ions at this concentration did not affect lipase activity of *Cupriavidus necator* H16 as much as 1 mM of metal ions (Lu et al. 2013). In this study, 5 out of 9 lipases were affected by 0.1 mM metal ions as shown by decreasing opacity of hydrolysis spots on film. On the other hand, there was not much study on the effect of 10 mM of metal ions on lipase activity. According to Abramic et al. 10 mM of Ca^2+^ slightly increased lipase activity of *Streptomyces rimosus* while Cu^2+^ at this concentration slightly reduced its lipase activity. By comparing to 1 mM, 10 mM of metal ions tend to reduce lipase activity (Abramić et al. 1999). However, in this study, 10 mM of metal ions did not influence the lipase activity on P(3HB-*co*-4HB) film. Thus, more studies should be carried out to investigate the reason behind this observation. Results from this study had shown that 1 mM of Cu^2+^ decreased or inhibited lipase activity of most tested lipases. Transition metal ions such as Cu^2+^ and Mn^2+^ have the ability to inhibit lipase activity because the ions will interact with enzyme surface charge that in turn reduce the enzyme stability and change its conformation (El-Rahman et al. 2006). On the other hand, Ca^2+^ and Mg^2+^ have the ability to stimulate lipase activity by altering the structure of catalytic site of lipase (Williams 1953). Apart from that, metal ions may act as stabilizers for tertiary lipase structure or activate water molecules for catalysis (Jaeger et al. 1999).

It is known that lipases act on the boundary of aqueous and oil interface. To improve the accessibility of both buffer and substrate, surfactants or emulsifiers are added. Detergents have been shown to cause protein aggregation because protein-detergent interactions are generally hydrophobic in nature (Møller and Le Maire 1993). In this study, it was also found that detergents are able to affect lipase activity on solid P(3HB-*co*-4HB) films. Four types of detergents were tested at different concentrations (0.01, 0.1 and 1 %). For detergents with 0.01 % of concentration, lipase activities of most lipases in this study were barely affected but lipase from *R*. *arrhizus* was significantly reduced. Most of the lipases were not affected by this concentration of detergents because lower concentration of detergents was suspected to have minimal influence on the lipases. Lipase activities from *C*. *antarctica* and *C*. *rugosa* was stimulated by 0.1 % of SDS, Tween 20 and Tween 80 while 0.1 % of Tween 20 and Tween 80 stimulated the lipase activities from *M*. *javanicus*. Lipase activities on P(3HB-*co*-4HB) film were inhibited after addition of 1 % detergents. This result suggested detergents at this concentration had exceeded its critical micelle concentration (CMC) and formed micelles (Moh’d and Wiegel 2010). This in turn inhibited lipase activity when the micelles were bound to the lipase lid and triggered change of lipase conformation (Reis et al. 2009). Addition of small amount of detergent is able to enhance lipase activity by changing the lipase’s conformation or interfacial properties (Reis et al. 2009). However, activities of other lipases on the film were mostly reduced or inhibited by detergents because detergents were able to compete with the active sites of lipases and therefore inhibit the lipase activities (Rathi et al. 2001; Quyen et al. 2005). Besides, detergents were found to form a protective layer on lipase substrate and in turn prevented accessibility of lipase towards substrates (Yao et al. 2013).

High production cost of PHA has lowered its value for common usage and more valuable applications are required to be developed. To date, there is no report for the screening of lipase activity on P(3HB-*co*-4HB) film using either medical or environmental samples. If lipase activity of medical or environmental samples could be detected and quantified using P(3HB-*co*-4HB) film, this copolymer film could be further developed as a standard lipase activity assay substrate that has a higher value compared to other applications such as production of plastic bags. In order to further determine the potential applications of the PHA film used in this study, extracts from mice and chicken organs were screened for depolymerizing activities on P(3HB-*co*-4HB) film. The idea was to determine whether there are lipase-like enzymes present in these organs that can produce hydrolysis spots on the films. Extracts obtained from organs of mouse and chicken were screened using P(3HB-*co*-4HB) film. Assay results have shown that hydrolysis spots were produced on the film by duodenum and pancreatic extracts of both animals. These two organs are known to contain one or more types of lipases (Carey et al. 1983) that may have the ability to degrade the film by forming opaque hydrolytic spots. The extract from gizzard in chicken was also able to form hydrolysis spots on the film. The gizzard is part of a chicken’s digestive tract and its function is similar to stomach for food digestion. Chicken fat was also collected and its extract formed hydrolysis spots on the film. However, the depolymerizing activities from gizzard and fat extracts were lower compared to those of duodenum and pancreas. Based on the results, the enzyme that was involved in this hydrolysis spots formation was most probably a type of lipase. This is the first report to prove that there are biological agents, which most probably are enzymes in animal organs that can hydrolyze P(3HB-*co*-4HB). Further studies are needed to identify these enzymes. Many studies have shown that PHA-based biomaterials undergo some form of hydrolysis when implanted into animal bodies (Chen and Wu 2005; Ying et al. 2008). However, the depolymerizing activities were not determined in those studies. The current study provides the first direct evidence for the presence of lipases and lipase-like enzymes which may play an important role in the in vivo degradation of PHA.

In order to confirm the presence of lipases in the crude organ extracts from both animals, their lipolytic activity were also screened using *p*NPL as substrate. This substrate is a synthetic ester that is formed by the esterification of *p*-nitrophenol and lauric acid which consists of 12 carbon atoms. Lipases are able to cleave the ester bond of *p*NPL (synthetic fatty acid esters) and release *p*-nitrophenol as a yellow colored end product. The amount of *p*-nitrophenol released can be quantified using spectrophotometer at a wavelength of 410 nm (Kilcawley et al. 2002). The lipase activities of duodenum from both animals’ organs assayed using this substrate was lower compared to other organs as shown in Figs. 6 and 7. This might be due to the substrate specificities of the lipase in each organ towards *p*-nitrophenyl laurate. The heated extracts showed lower or no activity with comparison to the buffer that did not cause degradation on the film surface and hydrolysis of *p*-nitrophenyl laurate. These supernatants were heated to denature the enzymes in the solutions so that they could act as negative control for the lipase activity assays either by using *p*NPL or P(3HB-*co*-4HB) as substrates. The lipase activities were different by using these two substrates because lipases in the organ extracts probably have different specificities towards these substrates (Gillmor et al. 1997). In conclusion, P(3HB-*co*-4HB) copolymer can be further developed as lipase depolymerizing activity assay substrate for applications in academic, industrial and medical fields.

## Additional file

10.1186/s13568-016-0230-z Additional materials.

## Abbreviations

P(3HB-*co*-4HB): poly(3-hydroxybutyrate-*co*-4-hydroxybutyrate)
PHA: polyhydroxyalkanoate
*p*NPL: *p*-nitrophenyl laurate
4HB: 4-hydroxybutyrate
3HP: 3-hydroxypropionate
5HV: 5-hydroxyvalerate
P(4HB): poly(4-hydroxybutyrate)
PBS: phosphate buffered saline
NB: nutrient broth
NM: nitrogen-free mineral medium
GC: gas chromatography
SEM: scanning electron microscope
QCM: quartz crystal microbalance
SDS: sodium dodecyl sulfate
PVA: polyvinyl alcohol
DMSO: dimethyl sulfoxide
GLM: general linear model
CMC: critical micelle concentration
