# Supplementary material for: Characterization of the depolymerizing activity of commercial lipases and detection of lipase-like activities in animal organ extracts using poly(3-hydroxybutyrate-co-4-hydroxybutyrate) thin film
Source: AMB Express. 2016 Oct 12;6:97. doi: 10.1186/s13568-016-0230-z (PMC5059232; doi:10.1186/s13568-016-0230-z)
Supplement: Supplementary file 1 — 10.1186/s13568-016-0230-z Additional materials. [file 13568_2016_230_MOESM1_ESM.docx]

**AMB Express**

**Characterization of the depolymerizing activity of commercial lipases and detection of lipase-like activities in animal organ extracts using poly(3-hydroxybutyrate-*co*-4-hydroxybutyrate) thin film**

Pei-Shze Mok^a^, Diana Hooi-Ean Ch’ng^a^, Soo-Peng Ong^a^, Keiji Numata^b^ and Kumar Sudesh*^a^

^a^ School of Biological Sciences, Universiti Sains Malaysia, 11800 Pulau Pinang, Malaysia.

^b^ Enzyme Research Team, RIKEN Center for Sustainable Resource Science, 2-1 Hirosawa, Wako-shi, Saitama 351-0198, Japan

* Corresponding author

Mailing address:

Kumar Sudesh

School of Biological Sciences,

Universiti Sains Malaysia,

11800 Penang,

Malaysia.

Tel: +60-4-6534367

Fax: +60-4-6565125

E-mail: ksudesh@usm.my

Table S1 Physical and thermal properties of P(3HB-*co*-4HB) (Saito et al*.*, 1996)

|  | 4HB fraction (mol %) | | | | | | | | | | |
| --- | --- | --- | --- | --- | --- | --- | --- | --- | --- | --- | --- |
|  | 0 | 3 | 7 | 10 | 16 | 27 | 64 | 78 | 82 | 90 | 100 |
| Melting temp (°C) | 178 |  | 172 |  | 130 |  | 50 | 49 | 52 | 50 | 53 |
| Glass transition temp (°C) | 4 |  | −2 |  | −7 |  | −35 | −37 | −39 | −42 | −48 |
| Crystallinity (%) | 60 | 55 | 50 | 45 | 45 | 40 | 15 | 17 | 18 | 28 | 34 |
| Density (g/cm^3^) | 1.250 |  |  | 1.232 | 1.234 | 1.234 |  |  |  |  |  |
| Water uptake (wt %) | 0.32 |  |  | 0.20 | 0.14 | 0.45 |  |  |  |  |  |
| Stress at yield (MPa) |  | 34 |  | 28 | 19 |  |  |  |  |  | 14 |
| Elongation at yield (%) |  | 4 |  | 5 | 7 |  |  |  |  |  | 17 |
| Tensile strength (MPa) | 43 | 28 |  | 24 | 26 |  | 17 | 42 | 58 | 65 | 104 |
| Elongation to break (%) | 5 | 45 |  | 242 | 444 |  | 591 | 1120 | 1320 | 1080 | 1000 |

Table S2 Sources and concentration used of commercial lipases (Sigma-Aldrich technical information)

| Lipase name | Product number | Lipase activity (U/mg) | Unit definition | Concentration used in this study (mg/mL) |
| --- | --- | --- | --- | --- |
| Lipase from*Candida antarctica* | 65986 | ≥1.0 | 1 U corresponds to the amount of enzyme which liberates 1 μmol oleic acid per min at pH 8.0 and 40 °C (triolein, Fluka No. 62314 as substrate) | 0.05 |
| Lipase from*Candida rugosa, Type VII* | L1754 | ≥700 | 1 U will hydrolyze 1.0 microequivalent of fatty acid from a triglyceride in 1 h at pH 7.2 at 37 °C | 1.00 |
| Amano Lipase M from *Mucor javanicus* | 534803 | ≥10 | - | 0.50 |
| Lipase from porcine pancreas, Type II | L3126 | 100-400 (using olive oil, 30 min incubation), 30-90 (using triacetin) | 1 U will hydrolyze 1.0 microequivalent of fatty acid from triacetin in 1 h at pH 7.4 at 37 °C. (pH 7.7 is used with olive oil as substrate) | 0.25 |
| Lipase from*Pseudomonas cepacia* | 62309 | ≥30 | 1 U corresponds to the amount of enzyme which liberates 1 μmol oleic acid per min at pH 8.0 and 40 °C (triolein, Fluka No. 62314 as substrate) | 0.25 |
| Amano Lipase from  *Pseudomonas fluorescens* | 534730 | ≥20 | - | 1.00 |
| Lipase from *Rhizopus arrhizus* | 62305 | ~10 | 1 U corresponds to the amount of enzyme which liberates 1 μmol of butyric acid per minute at pH 8.0 and 40 °C (tributyrin, Fluka No. 91010 as substrate); 5000 U as described above are equivalent to ~1 U using triolein, Fluka No. 62314 as substrate, at pH 8.0 and 40 °C | 0.12 |
| Lipase from *Rhizopus niveus* | 62310 | ≥1.5 | 1 U corresponds to the amount of enzyme which liberates 1 μmol fatty acid from a triglyceride per minute at pH 7.7 and 40 °C (olive oil as substrate)]; 300 U as described above are equivalent to ~1 U using triolein, Fluka No. 62314, at pH 8.0 and 40 °C as substrate | 4.00 |
| Lipase from *Rhizopus oryzae* | 80612 | ≥30 | 1 U corresponds to the amount of enzyme which releases 1 μmol fatty acid from triglycerides per minute at pH 7.2 and 37 °C (olive oil as substrate) | 0.25 |

Table S3 Compounds used for buffers preparation with different pH

| pH | Compounds | Volume (mL) |
| --- | --- | --- |
| 1 | 0.2 M KCl | 5.000 |
|  | 0.2 M HCl | 13.400 |
| 2 | 0.2 M KCl | 5.000 |
|  | 0.2 M HCl | 1.300 |
| 3 | 0.2 M Na_2_HPO_4_ | 2.040 |
|  | 0.1 M citric acid | 7.960 |
| 4 | 0.2 M Na_2_HPO_4_ | 3.860 |
|  | 0.1 M citric acid | 6.140 |
| 5 | 0.2 M Na_2_HPO_4_ | 5.140 |
|  | 0.1 M citric acid | 4.860 |
| 6 | 1 M K_2_HPO_4_ | 0.264 |
|  | 1 M KH_2_PO_4_ | 0.770 |
| 7 | 1 M K_2_HPO_4_ | 1.230 |
|  | 1 M KH_2_PO_4_ | 0.770 |
| 8 | 1 M K_2_HPO_4_ | 1.880 |
|  | 1 M KH_2_PO_4_ | 0.120 |
| 9 | 0.2 M glycine | 5.000 |
|  | 0.2 M NaOH | 0.880 |
| 10 | 0.05 M NaHCO_3_ | 10.000 |
|  | 0.1 M NaOH | 2.140 |
| 11 | 0.05 M NaHCO_3_ | 10.000 |
|  | 0.1 M NaOH | 4.540 |
| 12 | 0.05 M NaHCO_3_ | 10.000 |
|  | 0.1 M NaOH | 5.380 |
| 13 | 0.2 M KCl | 5.000 |
|  | 0.2 M NaOH | 13.200 |

The solutions were topped up to 20 mL.

**Figure captions**

**Fig. S1** Schematic diagram of degradation of amorphous part of P(3HB-*co*-4HB) film

**Fig. S2** Relationship of weight loss of P(3HB-*co*-4HB) film and relative density of opaque spot on P(3HB-*co*-4HB) film caused by lipase from a) *P*. *fluorescens* and b) *C*. *rugosa*

**Fig. S3** Hydrolysis spots of PBS pH 7.4 on P(3HB-*co*-4HB) film under different temperature from 15 °C to 60 °C. The assay was conducted for 30 minutes.

Hydrolysis spots were observed at 50, 55 and 60 °C indicating the occurrence of non-enzymatic hydrolysis

**Fig. S4** Depolymerizing activity assay on P(3HB-*co*-4HB) by extract from mice organs which are (b) duodenum, (c) duodenum*, (d) liver, (e) liver*, (f) spleen, (g) spleen*, (h) heart, (i) heart*, (l) pancreas, (m) pancreas*, (n) stomach, (o) stomach*, (p) lungs, (q) lungs*, (r) kidney, (s) kidney*. (a) and (k) indicate 0.25 mg/mL of lipase from *P*. *cepacia* which act as positive control while (j) and (t) indicate PBS which act as negative control. The assay was carried out at 37 °C for 1 hour. Each row of spots indicates triplicate. * indicates supernatant heated at 95 °C for 30 minutes

**Fig. S5** Depolymerizing activity assay on P(3HB-*co*-4HB) by extract from chicken organs which are (c) duodenum and pancreas, (d) duodenum and pancreas*, (e) fat, (f) fat*, (g) gizzard, (h) gizzard*, (k) liver, (l) liver*, (m) large intestine, (n) large intestine*, (o) small intestine, (p) small intestine*. (a) and (i) indicate 0.25 mg/mL of lipase from *P. cepacia* which act as positive control while (b) and (j) indicate PBS pH 7.4 which act as negative control. The assay was carried out at 37 °C for 1 hour. Each row of spots indicates triplicate. * indicates supernatant heated at 95 °C for 30 minutes

Degradation of amorphous part

Amorphous and crystalline parts of P(3HB-*co*-4HB) film that is transparent

Crystalline part of P(3HB-*co*-4HB) film that becomes opaque

**Fig. S1**

**Fig. S2**


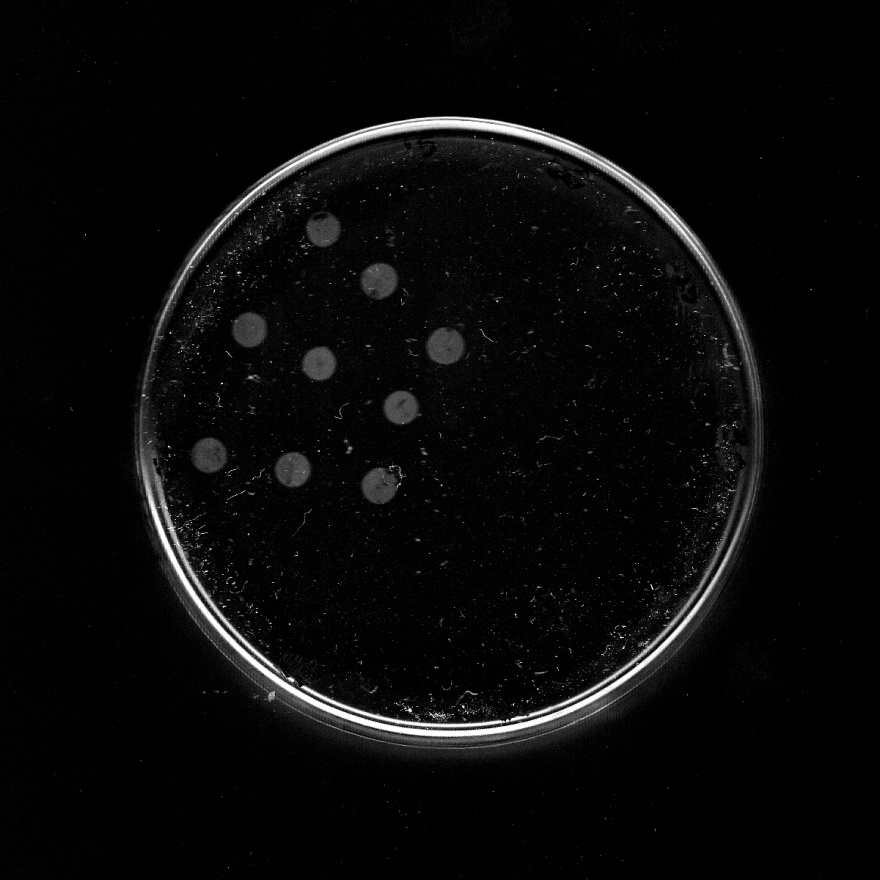


15 °C

25 °C

20 °C

30 °C

35 °C

40 °C

45 °C

50 °C

55 °C

60 °C

**Fig. S3**


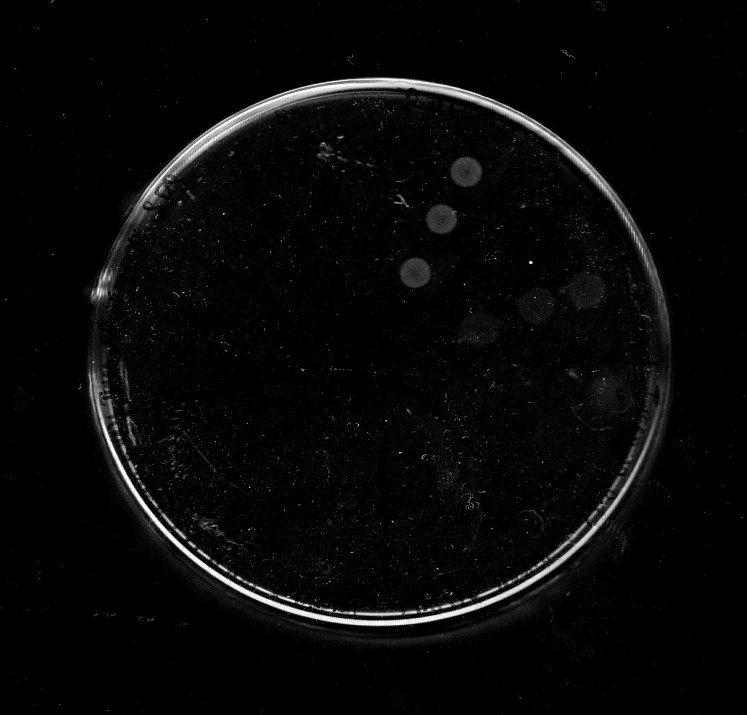

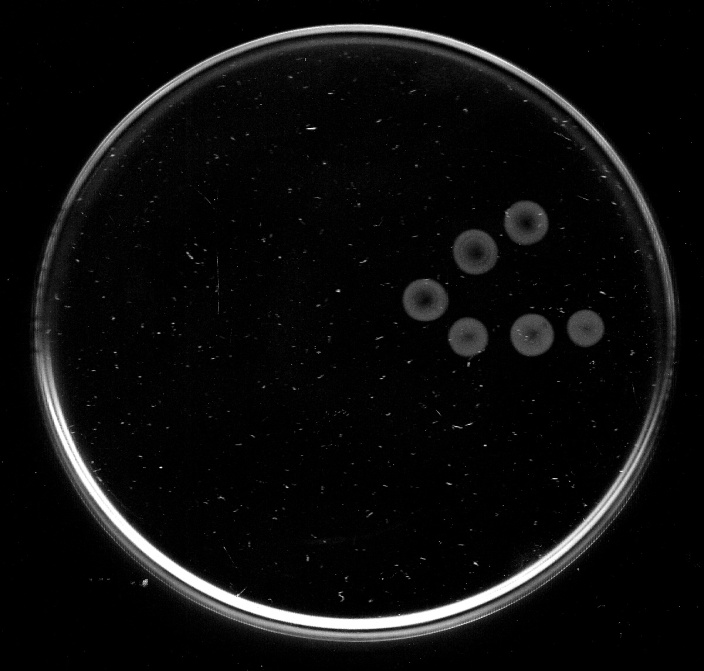


t

p

r

q

s

k

l

o

n

m

j

f

h

g

i

a

b

e

d

c

**Fig. S4**

**
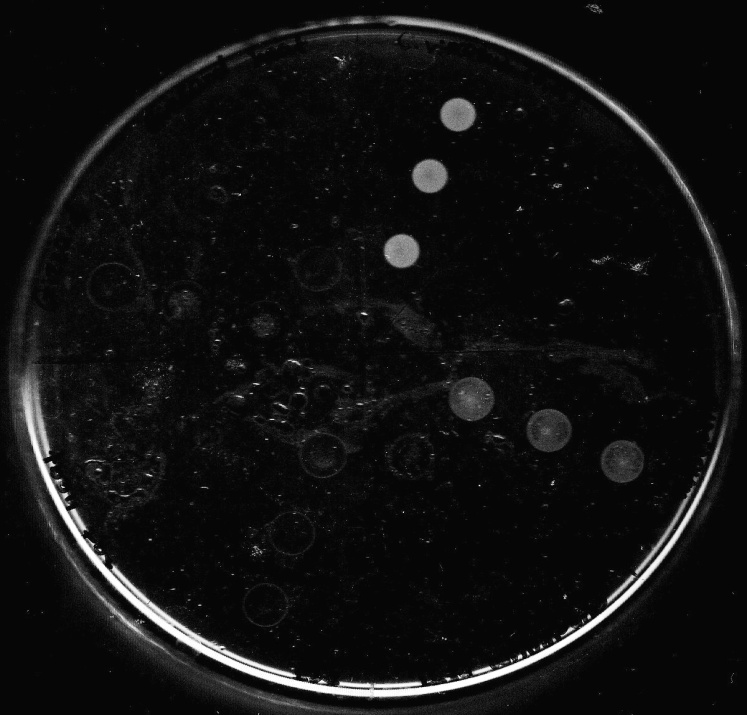
**
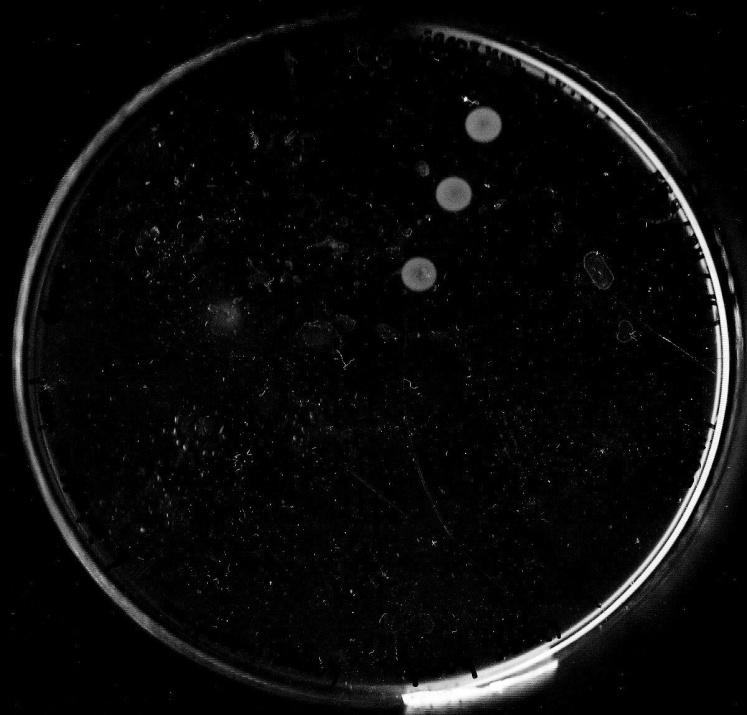


i

n

j

m

l

k

p

o

g

f

e

d

a

b

c

h

**Fig. S5**
